# Characterization of Clonixeril as a Positive Modulator of DMXAA-Induced Murine STING Signaling

**DOI:** 10.3390/biology15211979

**Published:** 2026-11-02

**Authors:** Anna Kharitonova, Rainer Metcalf, William Lawless, Rekha S. Patel, Bangmei Wang, Wayne C. Guida, Niketa A. Patel

**Affiliations:** 1 Research Service, James A. Haley Veteran’s Hospital, 13000 Bruce B Downs Blvd, Tampa, FL 33612, USA; kharitonova@usf.edu (A.K.); rekha.patel1@va.gov (R.S.P.); bangmei.wang@va.gov (B.W.); niketa.patel@va.gov (N.A.P.); 2 Department of Chemistry, University of South Florida, Tampa, FL 33620, USA; rainer@ashatherapeutics.com (R.M.); wlawless@usf.edu (W.L.); 3 Department of Molecular Medicine, University of South Florida, Tampa, FL 33612, USA

**Keywords:** STING, Clonixeril, DMXAA

## Abstract

Stimulator of Interferon Genes (STING) is a central mediator of innate immune signaling and a therapeutic target for inflammatory diseases. Previously, we identified Clonixeril (CXL) as a concentration-dependent inhibitory modulator of human STING (hSTING) signaling. Despite structural conservation, murine STING (mSTING) and hSTING differ in ligand recognition and activation. We posit that CXL may exhibit distinct activity in the murine system. Mice are used in biomedical research due to their genetic similarities to humans and their utility in evaluating systemic activity and safety of therapeutic compounds. We evaluated CXL effects on mSTING using computational modeling, as well as in vitro and in vivo studies. Molecular dynamics simulations and induced-fit docking predicted favorable CXL binding to mSTING. In 3T3-L1 cells, CXL enhanced DMXAA-induced STING, TBK1, and IRF3 phosphorylation and IFN-β mRNA expression, showing STING pathway activation. Acute CXL efficacy was evaluated in vivo in C57BL/6 mice. CXL increased IFN-β following DMXAA administration. Histopathology showed no treatment-associated abnormalities. HPLC detected CXL in serum and liver. The study identified species-specific responses, suggesting that mouse models may not accurately reflect the effects of CXL on hSTING signaling. Acute CXL treatment was well-tolerated, and CXL is a positive modulator of DMXAA-induced mSTING signaling in vivo.

## Simple Summary

STING protein is an important component of the immune system and has emerged as a promising therapeutic target for cancer, infectious diseases, and inflammatory disorders. Although human and murine STING share a similar overall structure, differences have been observed in the activity profiles of STING-targeting compounds. Mouse models provide an important platform for evaluating the systemic effects, therapeutic potential, and preliminary safety of new compounds in a physiological setting. We previously identified a small molecule called Clonixeril and demonstrated a concentration-dependent inhibitory effect on human STING. In this study, we examined the response to administering Clonixeril in murine models. Computational modeling and in vitro and in vivo studies demonstrated that Clonixeril enhances responses to the murine STING agonist DMXAA. Additionally, Clonixeril was well-tolerated under the conditions examined. These results demonstrate that Clonixeril potentiates DMXAA-induced murine STING signaling and emphasize the importance of accounting for species-specific differences during the development of STING-targeted therapeutics.

## 1. Introduction

Stimulator of Interferon Genes (STING) pathway is an essential mediator of innate immune responses, linking cytosolic DNA sensing to transcriptional activation of type I interferons (IFN-I) in both mice and humans following recognition of pathogen- or damage-associated nucleic acids. Dysregulation of STING signaling can disturb cellular and systemic homeostasis by promoting aberrant immune responses that contribute to the development and progression of various diseases [1,2]. Following detection of cytosolic double-stranded DNA, cyclic GMP–AMP synthase (cGAS) catalyzes production of the endogenous second messenger 2′,3′-cyclic GMP–AMP (2,3-cGAMP), the primary endogenous ligand of STING [3,4]. Binding of 2,3-cGAMP triggers conformational changes in STING that promote its dimerization, oligomerization, and trafficking from the endoplasmic reticulum to the Golgi apparatus. At the Golgi, STING recruits and activates TANK-binding kinase 1 (TBK1), which subsequently phosphorylates STING and Interferon Regulatory Factor 3 (IRF3). Phosphorylated IRF3 undergoes nuclear translocation, where it initiates transcription of IFN-I [5].

**Figure 1:**
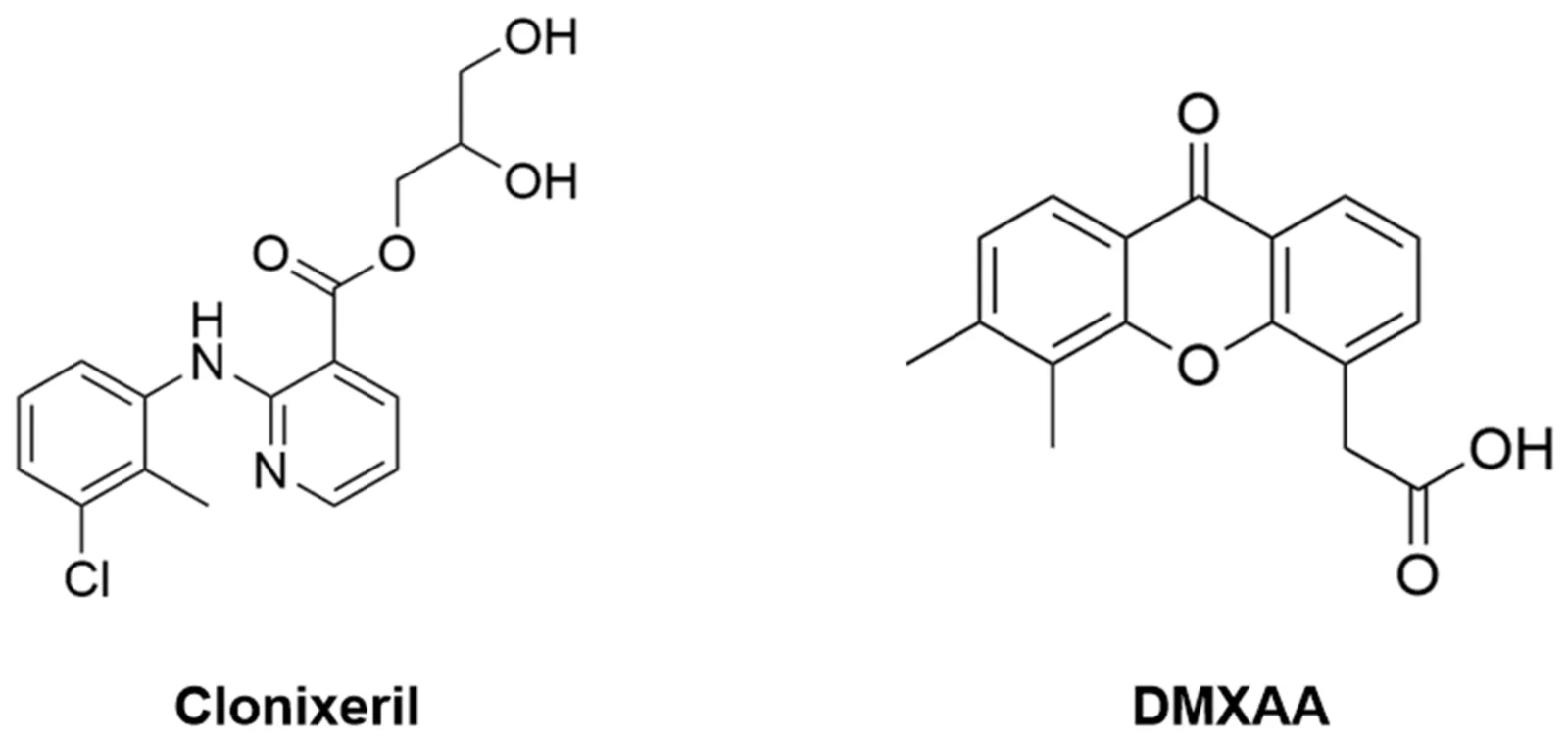
Chemical structures of Clonixeril (CXL) and DMXAA.

Previously, we identified Clonixeril (CXL) (Figure 1) through Molecular Dynamics (MD)-guided virtual screening as a non-nucleotide modulator of human STING (hSTING) [6]. Biophysical and cellular studies demonstrated that CXL exhibits concentration-dependent effects on STING signaling, functioning as a weak partial agonist at higher concentrations while suppressing hSTING activation at low concentrations. Previous data mechanistically infer that CXL interferes with STING oligomerization by stabilizing partially closed, autoinhibited oligomeric states that prevent productive downstream signaling. Consistent with this model, CXL reduced hSTING phosphorylation, IRF3 activation, and Interferon β (IFN-β) production in THP-1 and HEK293 cellular systems, while dynamic light scattering, mass photometry, microscale thermophoresis, and clear native PAGE experiments demonstrated significant effects on STING oligomerization. Collectively, these findings showed CXL modulated STING signaling through alteration of oligomerization dynamics rather than through simple competitive binding alone, motivating further investigation of its activity.

Although mSTING and hSTING are highly conserved, they differ markedly in ligand specificity, sensitivity, and receptor permissiveness. Consequently, mSTING responds to a broader range of endogenous and synthetic ligands, including DMXAA [7]. Given the widespread use of mouse models in preclinical research, understanding these species-specific differences is essential for evaluating the systemic activity, therapeutic potential, and preliminary safety of novel compounds in a physiological setting [8].

DMXAA (5,6-dimethylxanthenone-4-acetic acid) (Figure 1) is a synthetic flavonoid derivative that was initially developed as a vascular disrupting agent. Subsequent studies demonstrated that its biological activity results primarily from direct activation of mSTING. Upon binding mSTING with high affinity, DMXAA promotes closure of the STING dimer, initiates oligomerization, and robustly activates the TBK1–IRF3 signaling axis, leading to IFN-I production [7]. Unlike mSTING, hSTING is completely unresponsive to DMXAA, as steric hindrance and electrostatic incompatibility within the ligand-binding pocket preclude productive ligand binding, dimer stabilization, and downstream signaling [9]. The translational impact of this species-specific difference became evident in clinical trials, where DMXAA failed to show therapeutic benefit in human oncology despite its promising antitumor activity in murine models [10].

In view of the structural and functional differences between mSTING and hSTING, we investigated the activity of CXL in the mSTING model. We developed binding models for mSTING to predict binding modes and estimate the affinity of CXL for the cyclic dinucleotide-binding carboxy-terminus domain (CTD) using MD simulations and Induced Fit Docking (IFD). Downstream targets of mSTING signaling were evaluated in vitro using 3T3-L1 cells. Finally, the safety and efficacy of CXL were evaluated in a normal mouse model.

## 2. Materials and Methods

### 2.1. Computational Methodology

The mSTING computational model was generated from the PDB entry 4LOL containing DMXAA [11]; apo forms of mSTING and hSTING were taken from PDB entries 4KC0 and 4EMU, respectively. Each structure was prepared by removing crystallization additives such as sulfate and phosphate ions, bound ligands, and surplus protein dimers. Bond orders and disulfide bridges were assigned, and crystallographic hydrogens were stripped and regenerated to relieve steric clashes and other artifacts prior to protonation and hydrogen-bond optimization. Crystallographic waters were retained to inform side-chain protonation states and the initial hydrogen-bond network. Missing side chains were modeled and refined; hydrogens were added to the protein, remaining cofactors, and any modeled waters; and ionization states were predicted at pH 7.4 before a final round of hydrogen-bond optimization.

Molecular dynamics was carried out in Desmond version 7.9 using the OPLS-5 force field [12]. Systems were solvated with TIP3P water in a periodic box extending at least 10 Å beyond the protein surface and simulated at 310 K and 1 atm. Energy minimization was followed by a series of restrained minimizations to randomize starting configurations, and production trajectories of 250 ns were collected for every system.

Equilibration was judged from asymptotic behavior of the potential energy, root mean square deviation (RMSD), and radius of gyration (Rg), together with visual inspection of trajectories guided by root mean square fluctuation (RMSF) profiles. Once equilibrated, RMSD-based hierarchical clustering was applied to extract a representative average structure of mSTING.

Because both DMXAA and CXL occupy the STING-CTD site as dimers, binding affinities were estimated using the site-restricted virtual docking approach described by Sparks et al. [6]. Induced-fit docking (IFD) was layered onto this workflow to accommodate flexibility in both ligand and receptor [13]; the Schrödinger IFD protocol models conformational rearrangement of the binding site upon ligand binding, which improves affinity estimates and the prediction of candidate binding modes.

Two cubic grids were defined over the cyclic dinucleotide site, one centered on the centroid of the co-crystallized ligand and one on the centroid of the binding cavity. The receptor was minimized under constraints before an initial docking run using softened potentials. The 20 highest-scoring ligand poses were then used to sample receptor plasticity through conformational search and minimization of residues within 6 Å of any pose, and the resulting receptor conformations within 30 kcal/mol of the best-scoring complex were redocked.

### 2.2. Cell Culture

Mouse 3T3-L1 fibroblasts (ATCC^®^ CL-173™) were maintained at 37 °C under 10% CO_2_ in Dulbecco’s Modified Eagle’s Medium (Invitrogen, Carlsbad, CA, USA) containing 10% newborn calf serum (Sigma-Aldrich, St. Louis, MO, USA) and 1% penicillin–streptomycin (Gibco, Thermo Fisher Scientific, Waltham, MA, USA). Cultures were passaged before reaching confluence.

### 2.3. Western Blot Analysis

3T3-L1 cells were lysed in Cell Lysis Buffer (Cell Signaling Technology, Danvers, MA, USA; Cat. #9803S) containing protease and phosphatase inhibitor cocktails (Thermo Fisher Scientific, Waltham, MA, USA; Cat. #A32957 and #A32953). After brief sonication, protein concentrations were determined, and 50 μg per sample was resolved on 10% polyacrylamide gels and transferred to nitrocellulose membranes. Membranes were blocked in 5% nonfat dry milk in Tris-buffered saline containing 0.05% Tween-20 (TBST) and probed with antibodies against phospho-STING (Ser365) (clone D8F4W; Cell Signaling Technology, Danvers, MA, USA; Cat. No. 72971; 1:1000), total STING (clone D1V5L; Cell Signaling Technology, Danvers, MA, USA; Cat. No. 50494; 1:1000), phospho-TBK1 (Ser172) (clone D52C2; Cell Signaling Technology, Danvers, MA, USA; Cat. No. 5483; 1:1000), total TBK1 (Cell Signaling Technology, Danvers, MA, USA; Cat. No. 3013; 1:1000), phospho-IRF3 (Ser396) (clone 4D4G; Cell Signaling Technology, Danvers, MA, USA; Cat. No. 4947; 1:1000), total IRF3 (clone D83B9; Cell Signaling Technology, Danvers, MA, USA; Cat. No. 4302; 1:1000), and mouse monoclonal anti-β-actin (Sigma-Aldrich; Merck KGaA, St. Louis, MO, USA; Cat. No. A3854; 1:5000).

Membranes were subsequently incubated with species-appropriate horseradish peroxidase (HRP)-conjugated secondary antibodies (Bio-Rad Laboratories, Hercules, CA, USA) and developed with enhanced chemiluminescent substrate (Thermo Fisher Scientific; Cat. #32109). Images were captured on a ProteinSimple FluorChem™ (ProteinSimple, San Jose, CA, USA) system and quantified by densitometry in AlphaView software (version 3.4.0). Band intensities were normalized to β-actin, and each phosphoprotein was additionally expressed as a ratio to its corresponding total protein in the same lane (p-STING/STING, p-TBK1/TBK1, p-IRF3/IRF3). Only bands at the molecular weights indicated in the figures were quantified.

### 2.4. End-Point Polymerase Chain Reaction

Total RNA was isolated from 3T3-L1 cells using TRIzol™ Reagent (Thermo Fisher Scientific, Waltham, MA, USA; Cat. #15596026) according to the manufacturer’s protocol. RNA purity and concentration were assessed spectrophotometrically. Complementary DNA (cDNA) was synthesized from 1 μg RNA (260/230 > 1.8 and 260/280 > 1.8) using the iScript™ cDNA Synthesis Kit (Bio-Rad, Hercules, CA, USA; Cat. #1708891). End-point PCR amplification was performed using 1 μL cDNA template and JumpStart™ REDTaq^®^ ReadyMix™ Reaction Mix (Sigma-Aldrich, St. Louis, MO, USA; Cat. #P0982). Primer sequences used for amplification were as follows: β-actin (F: 5′-TGTCCACCTTCCAGCAGATGT-3′, R: 5′-AGCTCAGTAACAGTCCGCCTAGA-3′); IFN-β (F:’ 5′-GCCTTTGCCATCCAAGAGATGC-3′, R: 5′-ACACTGTCTGCTGGTGGAGTTC-3′). PCR products were separated on a 1% agarose gel, visualized using ethidium bromide, and imaged using the ProteinSimple FluorChem™ imaging system. Densitometric analysis was performed using AlphaView software.

### 2.5. Animal Study

All animal experiments were conducted under a protocol approved by the University of South Florida Institutional Animal Care and Use Committee (IACUC Protocol #IS00007192) and complied with the Animal Welfare Act (AWA), the Health Research Extension Act (HREA), and the ARRIVE guidelines. Male C57BL/6J mice (6–8 weeks old) were obtained from Jackson Laboratory and maintained in a specific pathogen-free facility under a 12 h light/12 h dark cycle with unrestricted access to food and water. Animals were housed in plastic cages containing sawdust bedding and allowed a two-week acclimation period before experimentation. Throughout the study, mice received a standard rodent chow diet (10 kcal% fat; Research Diets, Catalog #D12450Bi). Mice were randomly allocated into six groups (*n* = 6 per group). A two-step intraperitoneal (IP) dosing regimen was used. During the first stage, animals received either vehicle (5% ethanol solution) or CXL at doses of 1, 5, or 10 mg/kg body weight. One hour later, mice received a second IP injection consisting of either 5% sodium bicarbonate (vehicle control for DMXAA) or DMXAA (20 mg/kg). Group 1 received no injections and served as an untreated baseline control, whereas Group 2 received both vehicles on the same schedule and controlled for the effects of injection and vehicle administration. Five hours after DMXAA administration, animals were euthanized under CO_2_ anesthesia followed by cervical dislocation. Whole blood was collected by cardiac puncture for serum isolation, and tissues were harvested immediately and stored at −80 °C until further analysis.

### 2.6. Hematoxylin and Eosin Staining

Liver, spleen, and kidney were collected after perfusion and fixed for 24 h in 4% paraformaldehyde at 4 °C. Tissues were cryoprotected in 30% sucrose until equilibrated, embedded, and cut at 30–35 μm on a cryostat. Sections were mounted on gelatin-coated glass slides and held at 4 °C until staining. Hematoxylin and eosin staining followed the protocol supplied with a commercial kit (Abcam, Waltham, MA, USA; Cat. #ab245880), with staining times adjusted by tissue type (approximately 30 s to 2 min) to obtain adequate contrast. Stained sections were rinsed in distilled water, dehydrated through graded ethanol (70%, 95%, and 100%; 2 min each), cleared in xylene, and coverslipped with Permount mounting medium (Fisher Chemical, Pittsburgh, PA, USA; Cat. #SP15-500). Brightfield images were collected on a Keyence BZ-X810 digital microscope (Keyence Corporation of America, Itasca, IL, USA).

### 2.7. ELISA

Serum IFN-β was quantified with the Mouse IFN-β Quantikine ELISA Kit (R&D Systems, Minneapolis, MN, USA; Catalog #MIFNB0) according to the manufacturer’s protocol. Serum was diluted 1:2 in Calibrator Diluent RD5-5, and 50 μL of diluted sample, standard, or control was added to wells containing 50 μL of Assay Diluent RD1W. Plates were incubated for 2 h at room temperature on an orbital shaker (500 ± 50 rpm), washed four times with 400 μL of 1× Wash Buffer per well, and incubated for a further 2 h under the same conditions with 100 μL of HRP-conjugated anti-mouse IFN-β monoclonal antibody. After four additional washes, wells received 100 μL of Substrate Solution (Color Reagents A and B mixed in equal volumes) and were held for 30 min at room temperature in the dark before addition of 100 μL of Stop Solution. Absorbance at 450 nm was read immediately on a BioTek Synergy MX plate reader (BioTek Instruments Inc., Agilent Technologies, Winooski, VT, USA) using Gen5 software (v1.10).

Standards and samples were run in duplicate on a single plate. Concentrations were interpolated from a four-parameter logistic curve fitted to duplicate recombinant mouse IFN-β standards (1000–15.6 pg/mL, two-fold serial dilution) prepared in Calibrator Diluent RD5-5 with relative (1/Y^2^) weighting, and interpolated values were multiplied by the dilution factor of two. The kit range of 15.6–1000 pg/mL therefore corresponds to a reportable serum range of 31.2–2000 pg/mL. Samples were excluded from quantitative analysis if they interpolated below the lower limit of quantification, if the duplicate coefficient of variation exceeded 20%, or if absorbance exceeded that of the highest calibrator. Duplicate wells were averaged so that each animal contributed a single value.

Of the six animals per group, the number included in the quantitative analysis was five (DMXAA), four (1 mg/kg CXL+DMXAA), five (5 mg/kg CXL+DMXAA), and four (10 mg/kg CXL+DMXAA). Exclusions comprised three samples below the lower limit of quantification, one with a duplicate coefficient of variation exceeding 20%, one with absorbance exceeding the highest calibrator, and one identified as an outlier by Grubbs’ test. All criteria were defined and applied prior to statistical comparison.

### 2.8. High-Performance Liquid Chromatography

Tissue samples were extracted in 100% methanol and dried overnight under a gentle stream of nitrogen at room temperature. The dried extracts were reconstituted in equal volumes of 100% methanol prior to analysis. A 20 μL aliquot of each sample was injected onto a Phenomenex C18 100 Å column (250 × 10 mm, 5 μm) using a Shimadzu liquid chromatography system. Chromatographic separation was performed using mobile phase A (100% water) and mobile phase B (100% methanol). The system was equilibrated at 10% B prior to injection. The gradient program consisted of a hold at 10% B for the first 5 min, followed by a linear increase to 80% B from 5 to 35 min. Detection was carried out using a diode array detector scanning from 200 to 350 nm.

Homogenates containing both DMXAA and CXL were first extracted with ice-cold pure methanol to quench metabolism and recover total metabolites. The methanolic extracts were dried under nitrogen, reconstituted in water, and subjected to liquid–liquid partitioning with dichloromethane (DCM). This biphasic separation partitioned CXL into the aqueous layer and DMXAA into the organic (DCM) layer. Both phases were collected separately, evaporated to dryness, and reconstituted in methanol prior to HPLC analysis. All steps were performed at 4 °C or on ice to minimize degradation, with appropriate internal standards and quality controls incorporated for method validation.

### 2.9. Statistical Analysis

Statistical analyses were performed using GraphPad Prism version 11.1.0. Outliers were identified using Grubbs’ test (α = 0.05). Differences among groups were evaluated by one-way analysis of variance (ANOVA) followed by Tukey’s multiple-comparison test. Homogeneity of variance was assessed using the Brown–Forsythe test. Data are presented as mean ± SEM, and *p* < 0.05 was considered statistically significant.

## 3. Results

### 3.1. Computational Prediction of DMXAA and CXL Binding to mSTING

In hSTING, evidence suggests that CXL induces oligomers of insufficient length to support productive TBK1 and IRF3 complexation, precluding downstream IFN-β signal transduction [6]. STING-CTD LID destabilization appears key to this phenomenon, where CTD compaction from ligand binding reorients the CTD to allow oligomerization but fails to complete complexation when exposed to extremely low concentrations of CXL. Paradoxically, CXL is known to be a weak agonist at higher concentrations in hSTING and may have a similar modality for mSTING, acting as a potentiator instead of an inhibitor due to the unique mechanism of CXL. To assist in understanding this behavior, computational models were created and verified by reproducing known poses and yielding estimates consistent with reported experimental values for compounds DMXAA, CXL, and 2,3-cGAMP.

Molecular modeling using Site-Restricted IFD predicted CXL KD for mSTING of 290 nM, DMXAA at 240 nM, and 2,3-cGAMP at 160 nM. This is consistent with experimental K_D_ evaluation of DMXAA and 2,3-cGAMP binding to mSTING at 490 nM and 300 nM, respectively [14,15]. The computed KD of CXL for hSTING was 630 nM with an experimental K_D_ of 430 nM [6]. These benchmarks support the performance of the model in estimating CXL binding affinity for the mSTING CTD and in inferring induced conformational effects of ligand binding.

**Figure 2:**
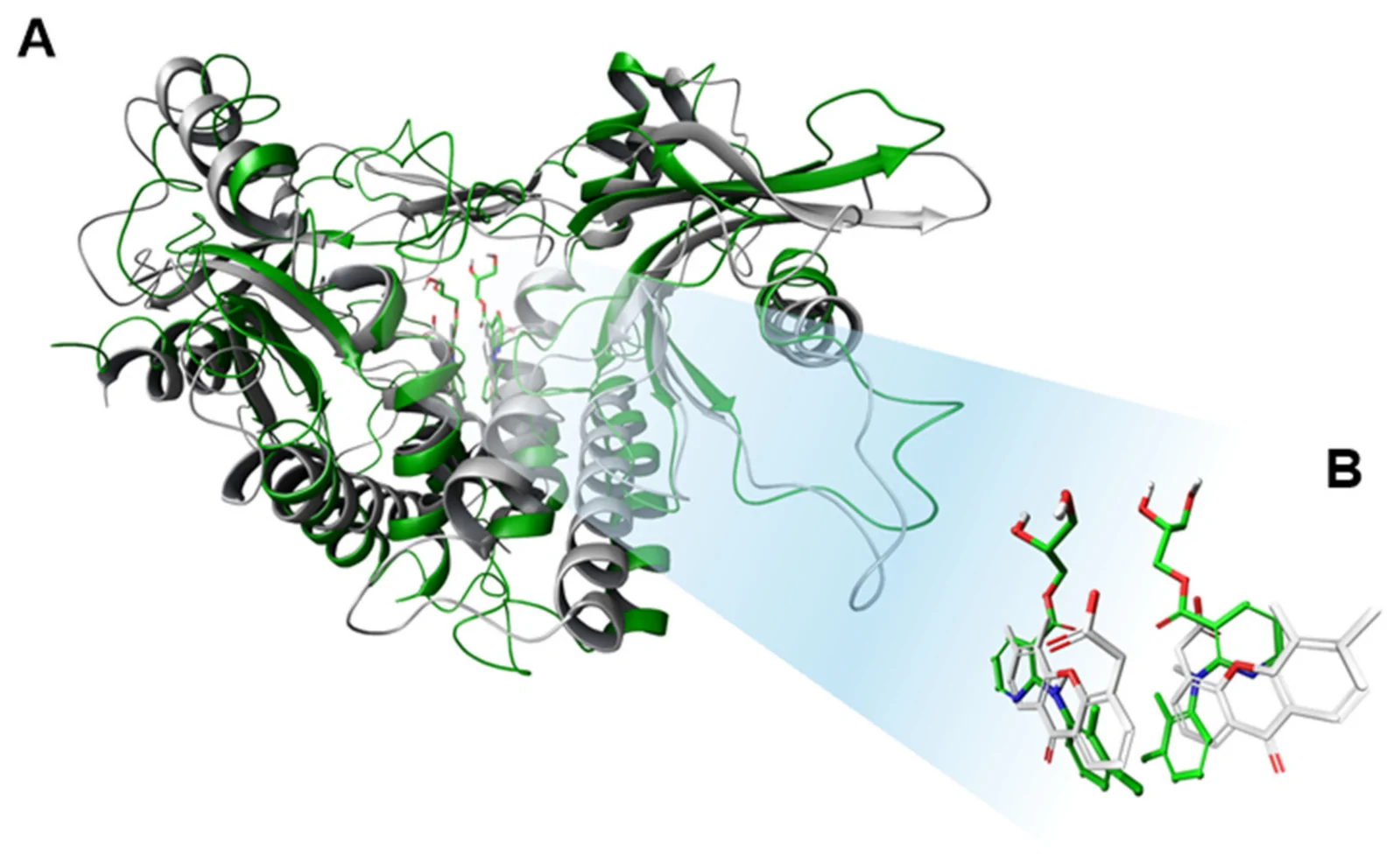
Site-restricted Induced Fit Docking (IFD) poses of murine Stimulator of Interferon Genes (mSTING) (**A**) with CXL (green ribbons) and DMXAA (gray ribbons). (**B**) Zoomed-in binding site overlay of CXL (green carbons) and DMXAA (gray carbons) molecular structures.

Binding pose overlays of DMXAA and CXL from simulations (Figure 2) show similar binding characteristics; however, CXL appears to interact with the STING LID region more than DMXAA. Our simulations support a model in which LID destabilization potentiates agonism in mSTING at low concentrations by inducing initial oligomerization, after which DMXAA is able to continue signal transduction. We propose that at higher concentrations, CXL competes with DMXAA for the binding pocket, moderating the combined response.

### 3.2. CXL Further Enhances DMXAA-Induced IFN-β mRNA Expression in 3T3-L1 Cells

**Figure 3:**
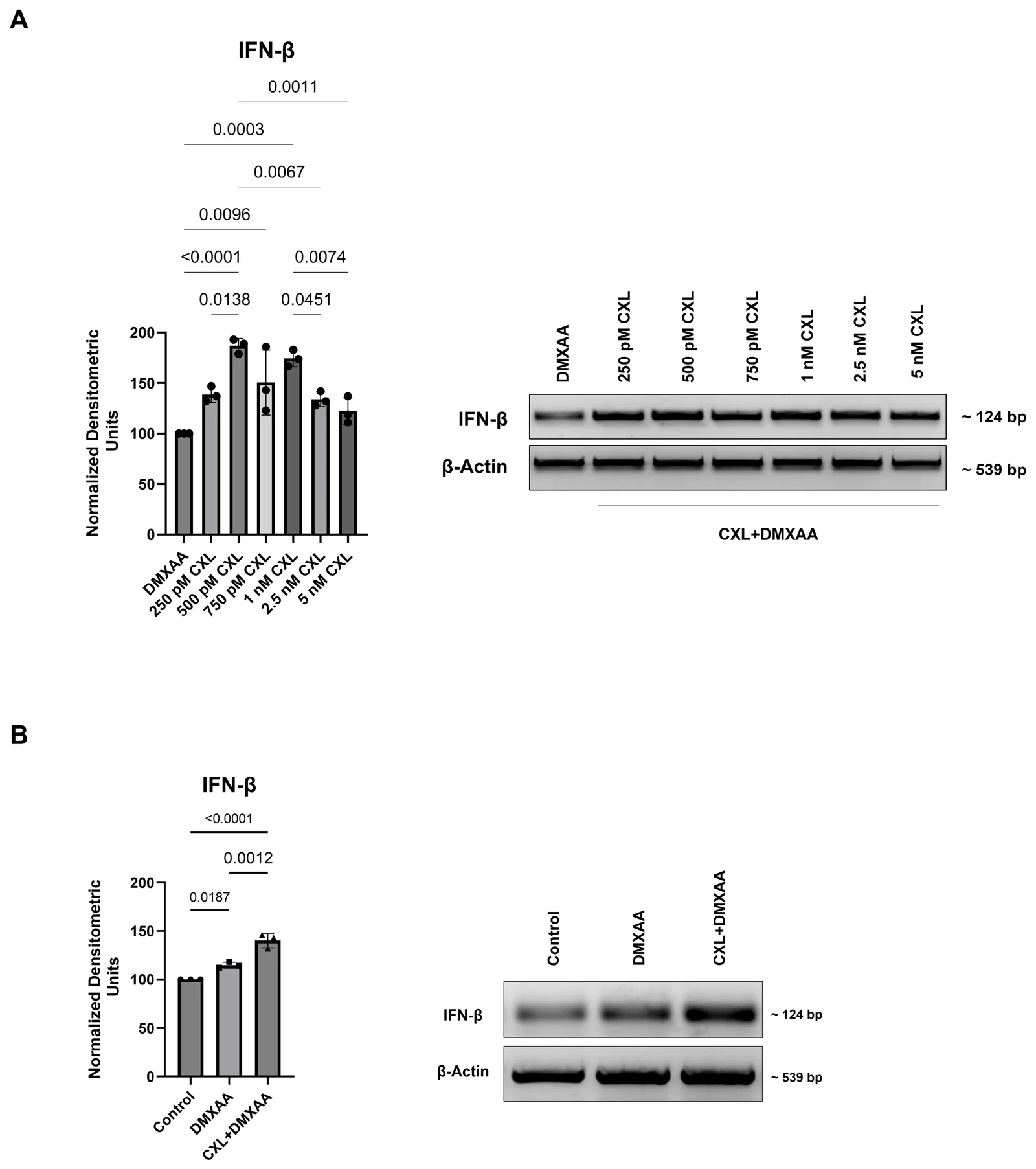
CXL Potentiates DMXAA-Induced Interferon β (IFN-β) mRNA expression in Murine 3T3-L1 Cells: (**A**) 3T3-L1 cells were seeded at 400,000 cells per 60 mm plate confluency. After 36 h, the control sample was treated with 5% NaHCO_3_; the CXL+DMXAA sample was treated with corresponding CXL concentrations (250 pM to 5 nM) for 1 h, followed by 50 μM DMXAA for 1 h and 30 min; and the DMXAA sample was treated with 50 μM DMXAA for 1 h and 30 min. RNA was extracted and used for end-point PCR analysis. Experiments were performed in three independent biological replicates, and representative gel images are shown. Images were digitally captured with ProteinSimple FluorChem™ and contrast-inverted for visualization purposes. Densitometric analysis was performed using AlphaView software. (**B**) 3T3-L1 cells were seeded at 400,000 cells per 60 mm plate confluency. After 36 h, the control sample was treated with 5% NaHCO_3_; the CXL+DMXAA sample was treated with 500 pM CXL for 1 h and then 50 μM DMXAA for 1 h and 30 min; and the DMXAA sample was treated with 50 μM DMXAA for 1 h and 30 min. RNA was extracted and used for end-point PCR analysis. Experiments were performed in three independent biological replicates, and representative gel images are shown. Images were digitally captured with ProteinSimple FluorChem™ and contrast-inverted for visualization purposes. Densitometric analysis was performed using AlphaView software. Data represent mean ± SEM. Statistical analysis was performed using one-way ANOVA followed by Tukey’s multiple-comparison test in GraphPad Prism. Exact *p*-values are shown above each comparison in (**B**); *p*-values for comparisons with the DMXAA group in (**A**) are reported in the text.

3T3-L1 cells were selected as a well-characterized murine line for preliminary validation of CXL activity in a mouse system prior to the in vivo studies. To first evaluate the effects of CXL on mSTING signaling, IFN-β mRNA expression levels were assessed following treatment with DMXAA and treatment with CXL prior to DMXAA administration (Figure 3; Figure S1). IFN-β is a downstream effector of STING pathway activation and was evaluated as a functional marker of pathway signaling. To identify the CXL concentration for subsequent experiments, a concentration screen was performed. 3T3-L1 cells were pretreated with CXL at concentrations ranging from 250 pM to 5 nM for 1 h prior to stimulation with 50 μM DMXAA for 1 h and 30 min (Figure 3A). CXL pretreatment increased IFN-β mRNA levels relative to the DMXAA group, reaching significance at 500 pM (*p* < 0.0001), 750 pM (*p* = 0.0096), and 1 nM (*p* = 0.0003). The effect was most pronounced at 500 pM, which was therefore selected for subsequent experiments. 3T3-L1 cells were then treated with 50 μM DMXAA for 1 h and 30 min or pretreated with 500 pM CXL for 1 h prior to 50 μM DMXAA for 1 h and 30 min. DMXAA treatment increased IFN-β mRNA levels relative to the untreated control group, and treatment with CXL prior to DMXAA administration further increased IFN-β mRNA levels compared with the DMXAA group (Figure 3B).

### 3.3. CXL Potentiates DMXAA-Induced STING, TBK1 and IRF3 Phosphorylation in Murine 3T3-L1 Cells

**Figure 4:**
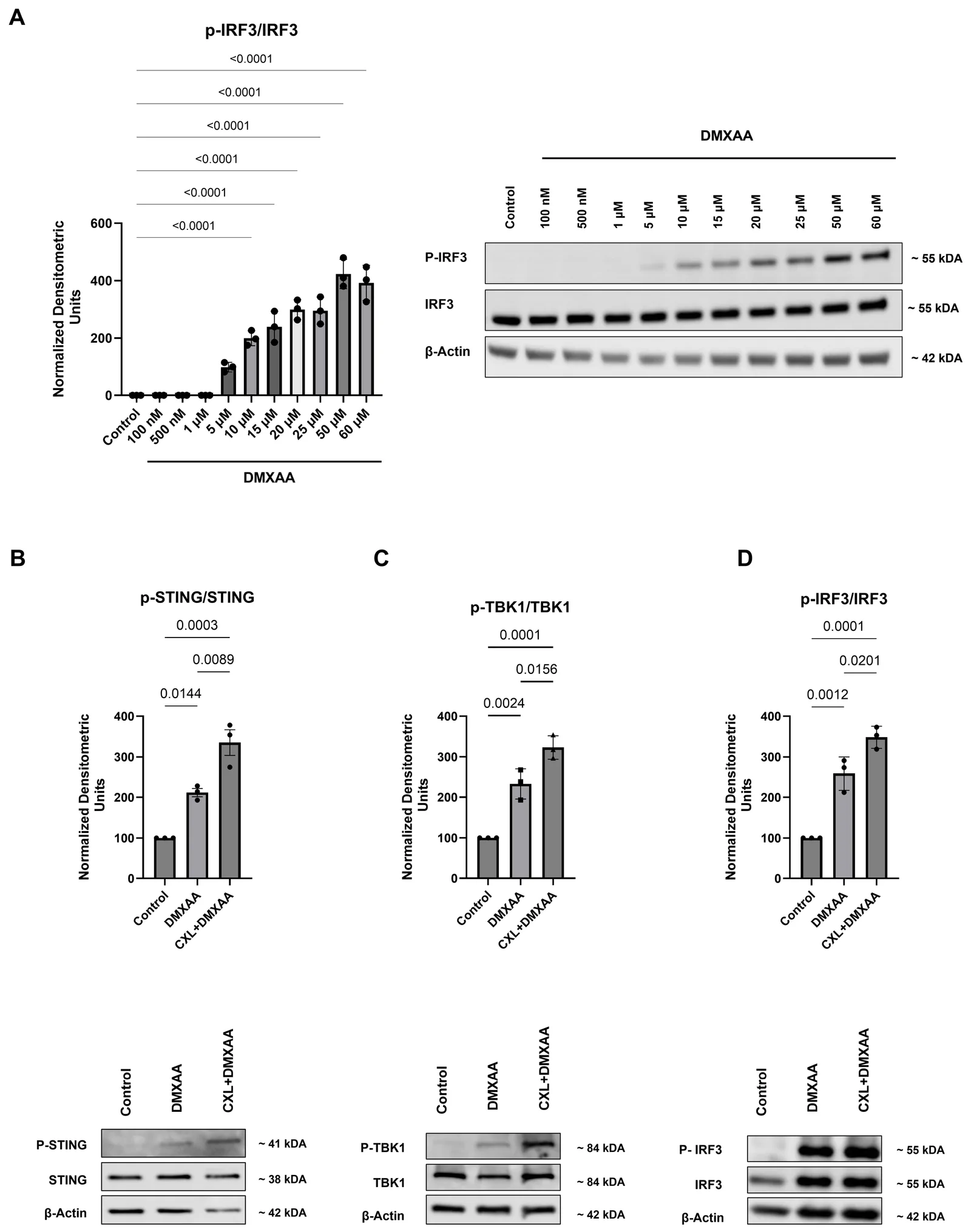
Effects of DMXAA and CXL on Stimulator of Interferon Genes (STING), TANK-binding kinase 1 (TBK1), and Interferon Regulatory Factor 3 (IRF3) phosphorylation in murine 3T3-L1 cells: (**A**) 3T3-L1 cells were seeded at 400,000 cells per 60 mm plate confluency. After 36 h, sample 1 was treated with 5% NaHCO_3_ (vehicle control), and samples 2–11 were treated with corresponding concentrations of DMXAA. Protein lysates were collected for Western blot analysis. Experiments were performed in three independent biological replicates, and representative blots are shown. Band intensities were normalized to β-actin, and p-IRF3 is expressed as a ratio to total IRF3. (**B**) 3T3-L1 cells were seeded at 400,000 cells per 60 mm plate confluency. After 36 h, samples 1 and 2 were treated with 5% NaHCO_3_ (vehicle control) and 50 μM DMXAA, respectively, for 1 h and 30 min. CXL+DMXAA samples were treated with 500 pM CXL for 1 h and then 50 μM DMXAA for 1 h and 30 min. Protein lysates were collected for Western blot analysis. Experiments were performed in three independent biological replicates, and representative blots are shown. Band intensities were normalized to β-actin, and p-STING is expressed as a ratio to total STING. (**C**) 3T3-L1 cells were seeded at 400,000 cells per 60 mm plate confluency. After 36 h, samples 1 and 2 were treated with 5% NaHCO_3_ (vehicle control) and 50 μM DMXAA, respectively, for 1 h and 30 min. CXL+DMXAA samples were treated with 500 pM CXL for 1 h and then 50 μM DMXAA for 1 h and 30 min. Protein lysates were collected for Western blot analysis. Experiments were performed in three independent biological replicates, and representative blots are shown. Band intensities were normalized to β-actin, and p-TBK1 is expressed as a ratio to total TBK1. (**D**) 3T3-L1 cells were seeded at 400,000 cells per 60 mm plate confluency. After 36 h, samples 1 and 2 were treated with 5% NaHCO_3_ (vehicle control) and 50 μM DMXAA, respectively, for 1 h and 30 min. CXL+DMXAA samples were treated with 500 pM CXL for 1 h and then 50 μM DMXAA for 1 h and 30 min. Protein lysates were collected for Western blot analysis. Experiments were performed in three independent biological replicates, and representative blots are shown. Band intensities were normalized to β-actin, and p-IRF3 is expressed as a ratio to total IRF3. Data represent mean ± SEM. Statistical analysis was performed using one-way ANOVA followed by Tukey’s multiple-comparison test in GraphPad Prism. Exact *p*-values are shown above each comparison.

Having observed that CXL pretreatment enhanced DMXAA-induced IFN-β mRNA expression, we next examined whether this effect was accompanied by changes in activation of the upstream STING signaling cascade. Accordingly, p-STING, p-TBK1, and p-IRF3 levels were assessed in 3T3-L1 cells. An initial dose–response analysis was performed to characterize DMXAA-induced IRF3 phosphorylation and establish the DMXAA concentration for subsequent experiments (Figure 4A; Figure S1). The concentration-dependent response confirmed functional mSTING signaling in this model, consistent with the established mSTING selectivity of DMXAA [7]. Based on these findings, 50 μM DMXAA was selected. Then, 3T3-L1 cells were treated with 50 μM DMXAA for 1 h and 30 min or pretreated with 500 pM CXL for 1 h before stimulation with 50 μM DMXAA for 1 h and 30 min (Figure 4B–D). DMXAA treatment increased p-STING, p-TBK1, and p-IRF3 levels compared with the untreated control group. The CXL+DMXAA group further enhanced DMXAA-induced STING, TBK1, and IRF3 phosphorylation compared with the DMXAA group.

### 3.4. CXL Further Enhances DMXAA-Induced IFN-β Production in C57BL/6 Mice

We evaluated biological effects of CXL on mSTING signaling in vivo using a two-stage intraperitoneal (IP) injection protocol in male C57BL/6 mice (6–8 weeks old; *n* = 6 per group) (Table 1). Mice received CXL vehicle control or CXL at doses of 1, 5, or 10 mg/kg. One hour later, mice received a second IP injection consisting of either DMXAA vehicle control (5% sodium bicarbonate) or DMXAA (20 mg/kg). This experimental design was intended to permit pharmacologic interaction of CXL with mSTING prior to pathway activation by DMXAA.

**Figure 5:**
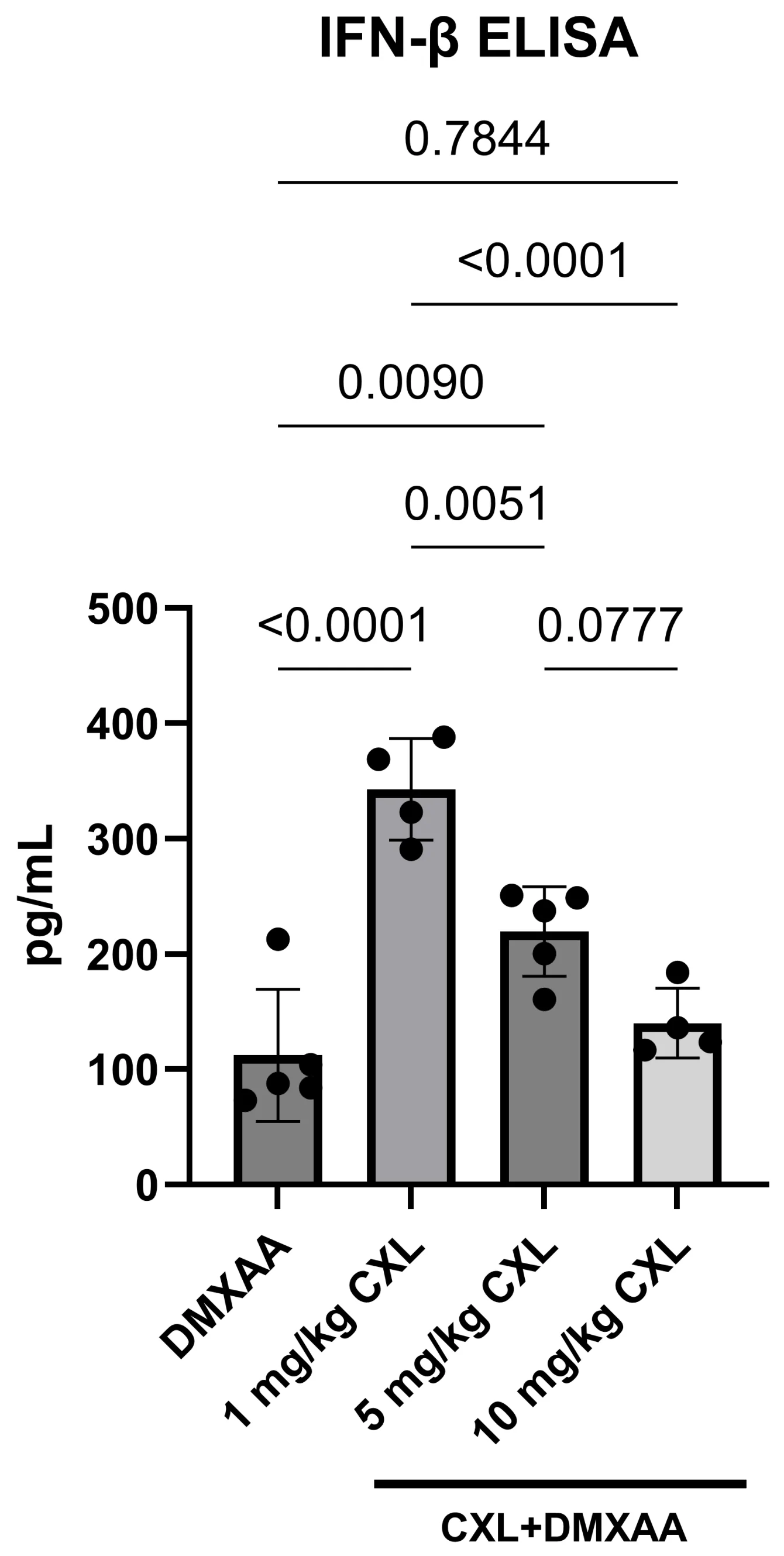
CXL enhances DMXAA-induced IFN-β production in C57BL/6 mice. Mice were pre-treated with CXL at 1, 5, or 10 mg/kg via IP injection one hour prior to administration of DMXAA (20 mg/kg). Five hours following DMXAA injection, blood was collected via terminal cardiac puncture, and serum IFN-β levels were measured using the Quantikine Mouse IFN-β Immunoassay (R&D Systems, Catalog #MIFNB0). IFN-β concentrations are shown in pg/mL. Data represent mean ± SEM (*n* = 4–5); individual animal values are shown as data points. Statistical analysis was performed using one-way ANOVA followed by Tukey’s multiple-comparison test in GraphPad Prism. Exact *p*-values are shown above each comparison.

To assess STING pathway activation in vivo, circulating levels of IFN-β were measured in blood serum from C57BL/6 mice following intraperitoneal administration of DMXAA and CXL+DMXAA (Figure 5). Serum was separated and analyzed using a quantitative IFN-β ELISA. Serum IFN-β was below the lower limit of quantification (31.2 pg/mL) in untreated and vehicle-treated groups, consistent with the absence of detectable IFN-β reported for normal mouse serum with this assay. In contrast, IFN-β was quantifiable in the DMXAA group (112.5 ± 25.8 pg/mL). The 1 mg/kg b.w. CXL+DMXAA group showed a 3.05-fold increase relative to DMXAA alone (343.0 ± 21.9 pg/mL; *p* < 0.0001). The 5 mg/kg b.w. CXL+DMXAA group showed a 1.95-fold increase (219.6 ± 17.2 pg/mL; *p* = 0.0090), whereas the 10 mg/kg b.w. CXL+DMXAA group (140.4 ± 15.2 pg/mL) did not differ significantly from DMXAA alone.

### 3.5. Histopathological Analysis Reveals No Treatment-Associated Changes

**Figure 6:**
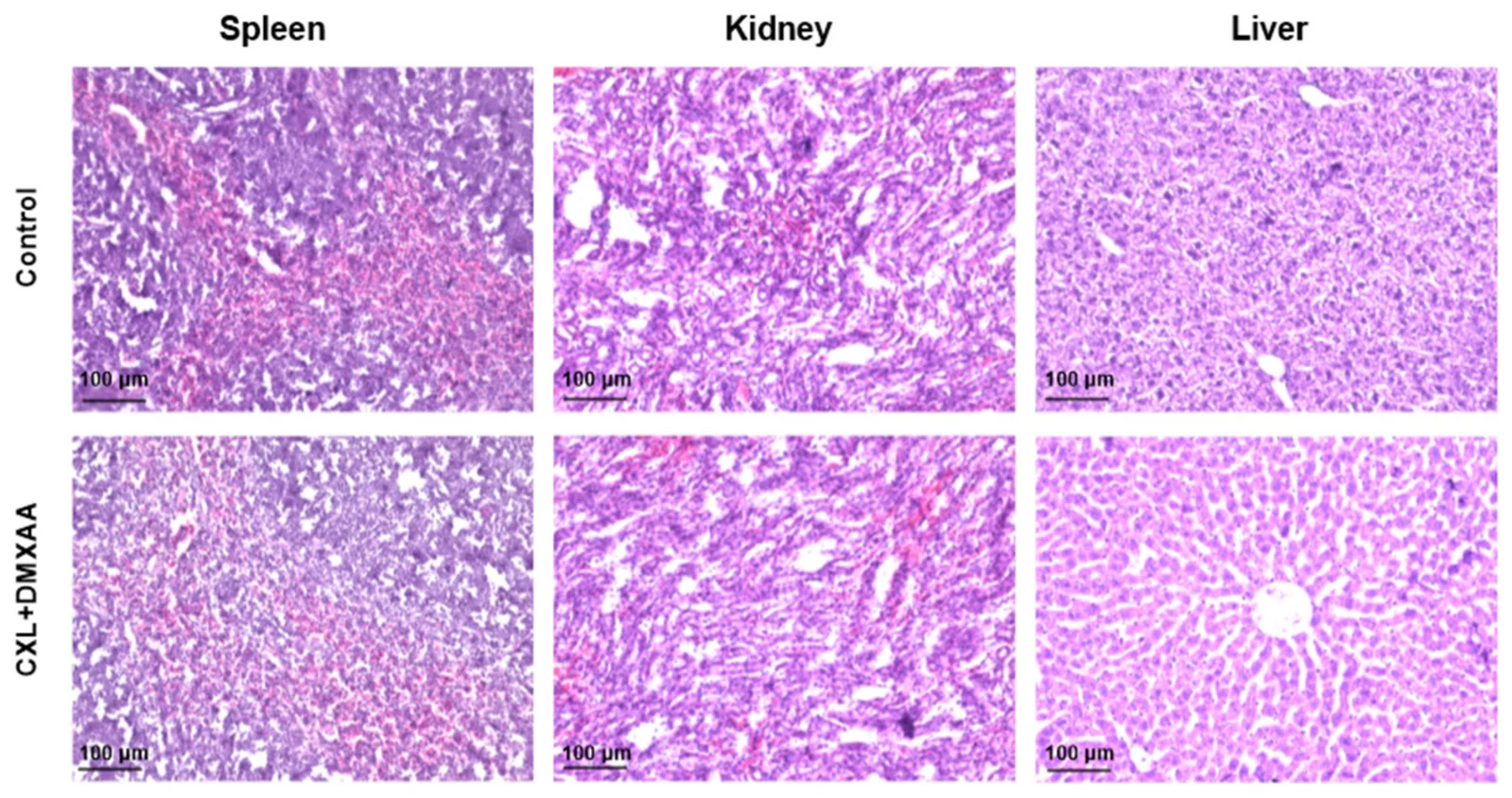
Histological evaluation following CXL+DMXAA treatment. Representative hematoxylin and eosin (H&E)-stained sections of spleen, kidney, and liver tissues from the control and CXL+DMXAA-treated groups (10 mg/kg b.w. CXL and 20 mg/kg b.w. DMXAA) are shown.

To evaluate the safety of CXL, histological analysis was performed using hematoxylin and eosin (H&E) staining of spleen, kidney, and liver tissues from the control group and the group treated with the highest dose of 10 mg/kg b.w. CXL and 20 mg/kg b.w. DMXAA. No changes in body weight, food intake, or behavior were observed during the treatment period. Across all examined tissues, histological evaluation (Figure 6) demonstrated preserved architecture, with no evidence of treatment-associated changes. All tissues exhibited normal histological features without signs of structural disruption, inflammation, or pathological alterations.

### 3.6. Renal Tissue Analysis Reveals No Detectable CXL Following Systemic Administration

**Figure 7:**
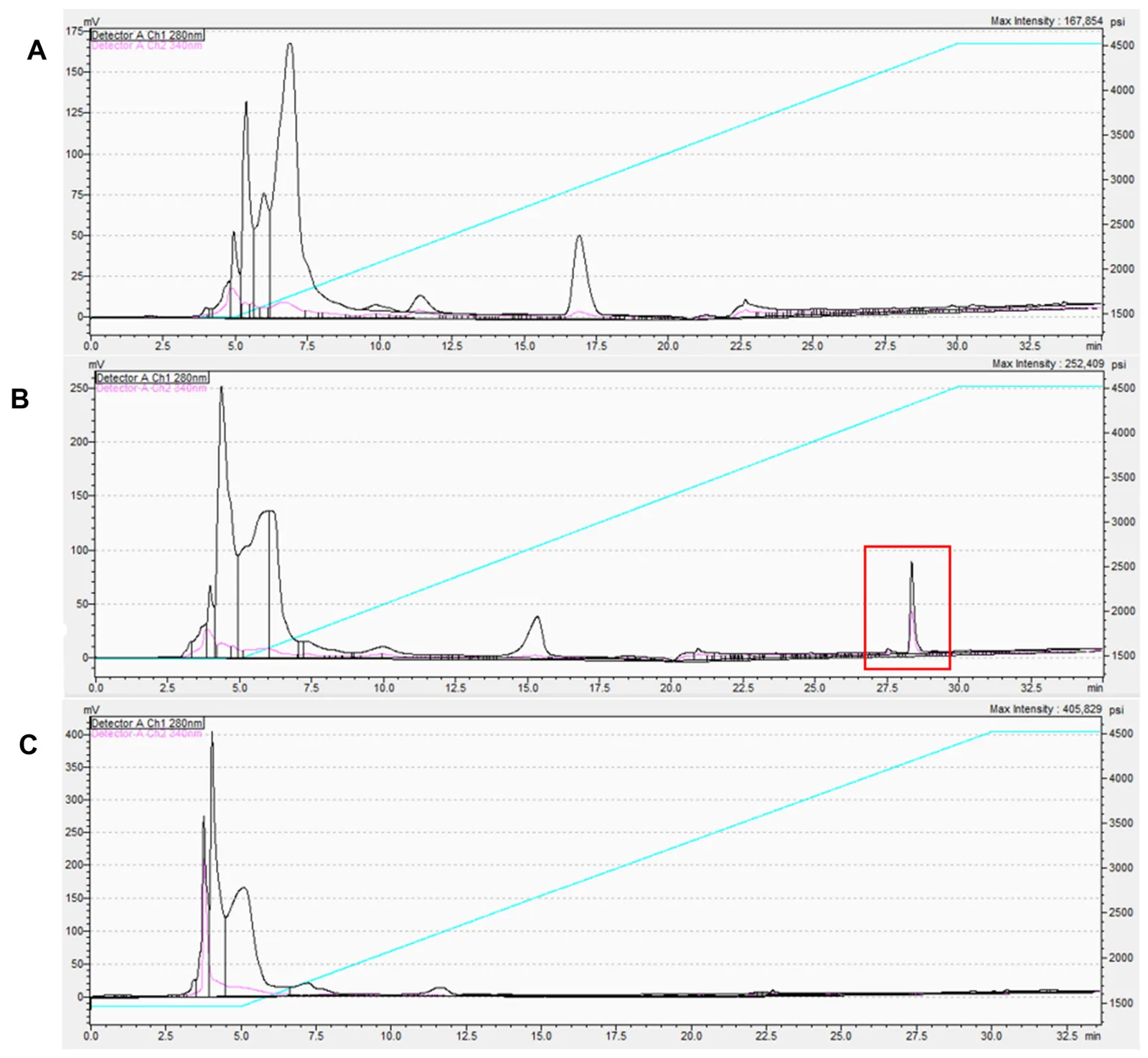
CXL is not detected in renal tissue following systemic administration. HPLC was performed on kidney tissue to evaluate the presence of CXL: (**A**) Chromatogram of untreated kidney tissue, serving as the negative control, shows no peak at the expected CXL retention time. (**B**) Chromatogram of kidney tissue spiked ex vivo with CXL reveals a distinct peak (highlighted), confirming the retention time and peak identity of CXL. (**C**) Chromatogram of renal tissue collected from mice treated in vivo with 10 mg/kg CXL shows no detectable CXL peak. The black trace represents detector channel 1 (280 nm) and the pink trace represents detector channel 2 (340 nm), both plotted against the left axis (mV). The cyan trace represents system backpressure, plotted against the right axis (psi).

The liver and kidneys are major organs involved in drug metabolism and elimination, while the spleen is an immune organ enriched in cell populations involved in innate immune signaling [16,17]. Thus, we examined whether CXL was present in these organs following systemic administration. Kidney samples collected 6 h after CXL IP administration of 10 mg/kg b.w. were analyzed by HPLC (Figure 7). Chromatographic analysis of untreated kidney homogenates showed no peak at the established retention time for CXL (Figure 7A). To confirm assay performance, untreated kidney homogenates were spiked ex vivo with CXL and used as positive controls. The spiked samples produced a distinct peak at 29 min, confirming reliable detection of CXL within the kidney tissue (Figure 7B). In contrast, chromatograms from kidneys collected from CXL-treated mice showed no detectable peak at the corresponding retention time (Figure 7C). Collectively, these findings indicate that CXL was not detectable in renal tissue 6 h after CXL administration under the conditions examined.

### 3.7. CXL Is Undetectable in Spleen Tissue Following Systemic Administration

**Figure 8:**
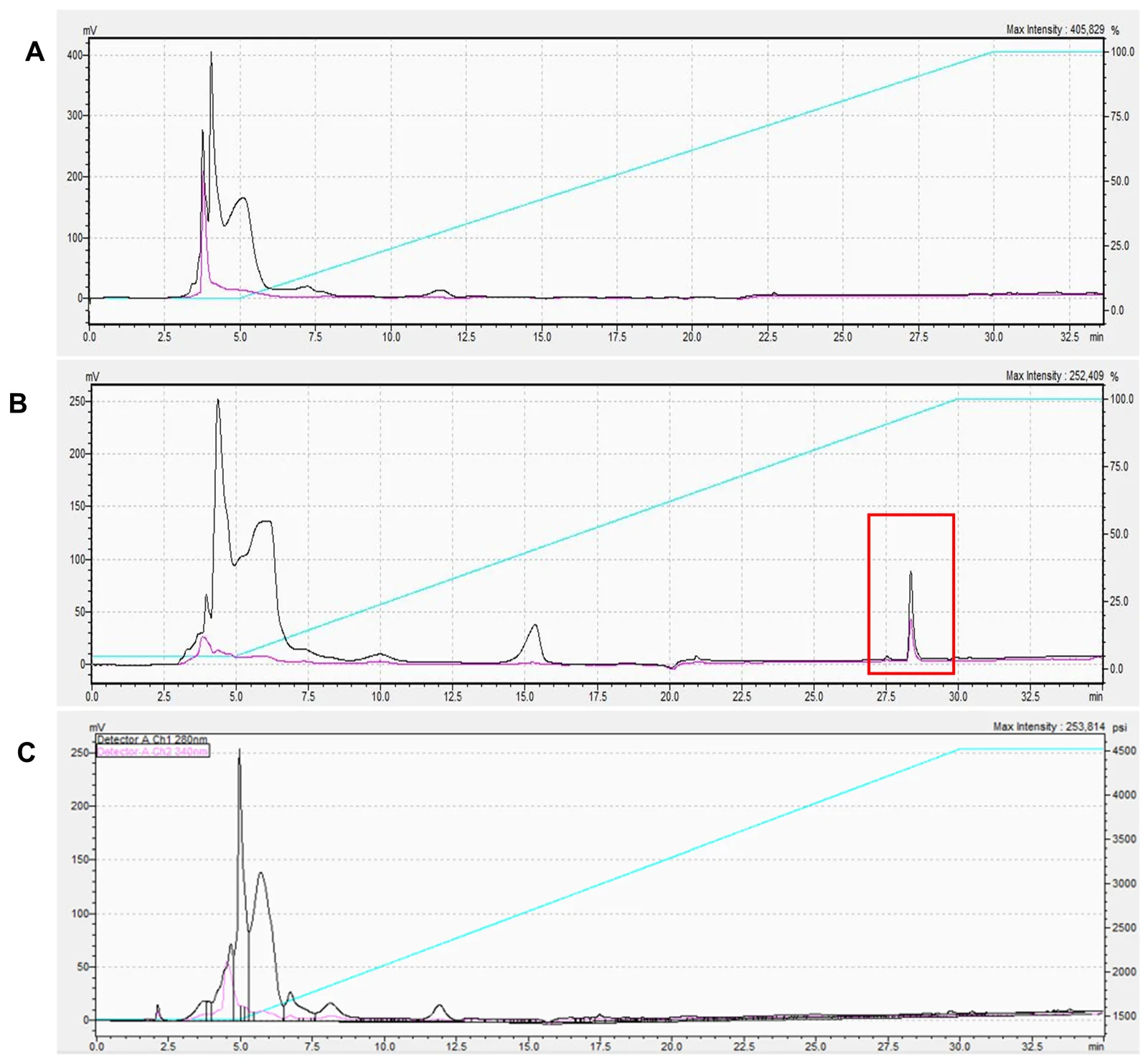
CXL is undetectable in spleen tissue following systemic administration. HPLC was performed on spleen tissue to evaluate the presence of CXL: (**A**) Chromatogram of untreated spleen tissue, serving as the negative control, shows no peak at the expected CXL retention time. (**B**) Chromatogram of spleen tissue spiked ex vivo with CXL reveals a distinct peak (highlighted), confirming the retention time and peak identity of CXL. (**C**) Chromatogram of spleen tissue collected from mice treated in vivo with 10 mg/kg CXL shows no detectable CXL peak. The black trace represents detector channel 1 (280 nm) and the pink trace represents detector channel 2 (340 nm), both plotted against the left axis (mV). The cyan trace represents system backpressure, plotted against the right axis (psi).

HPLC analysis was performed to determine whether CXL was present in splenic tissue 6 h after intraperitoneal administration of 10 mg/kg b.w. CXL (Figure 8). Untreated spleen homogenates exhibited no signal at the established CXL retention time (Figure 8A). Following ex vivo spiking with CXL, a distinct chromatographic peak was observed around the 29 min mark, confirming accurate detection of CXL within the spleen tissue (Figure 8B). No corresponding peak was detected in spleen samples obtained from CXL-treated mice (Figure 8C), indicating that CXL was not detectable in the spleen at this time point.

### 3.8. CXL Remains Detectable in Serum Following Systemic Administration

**Figure 9:**
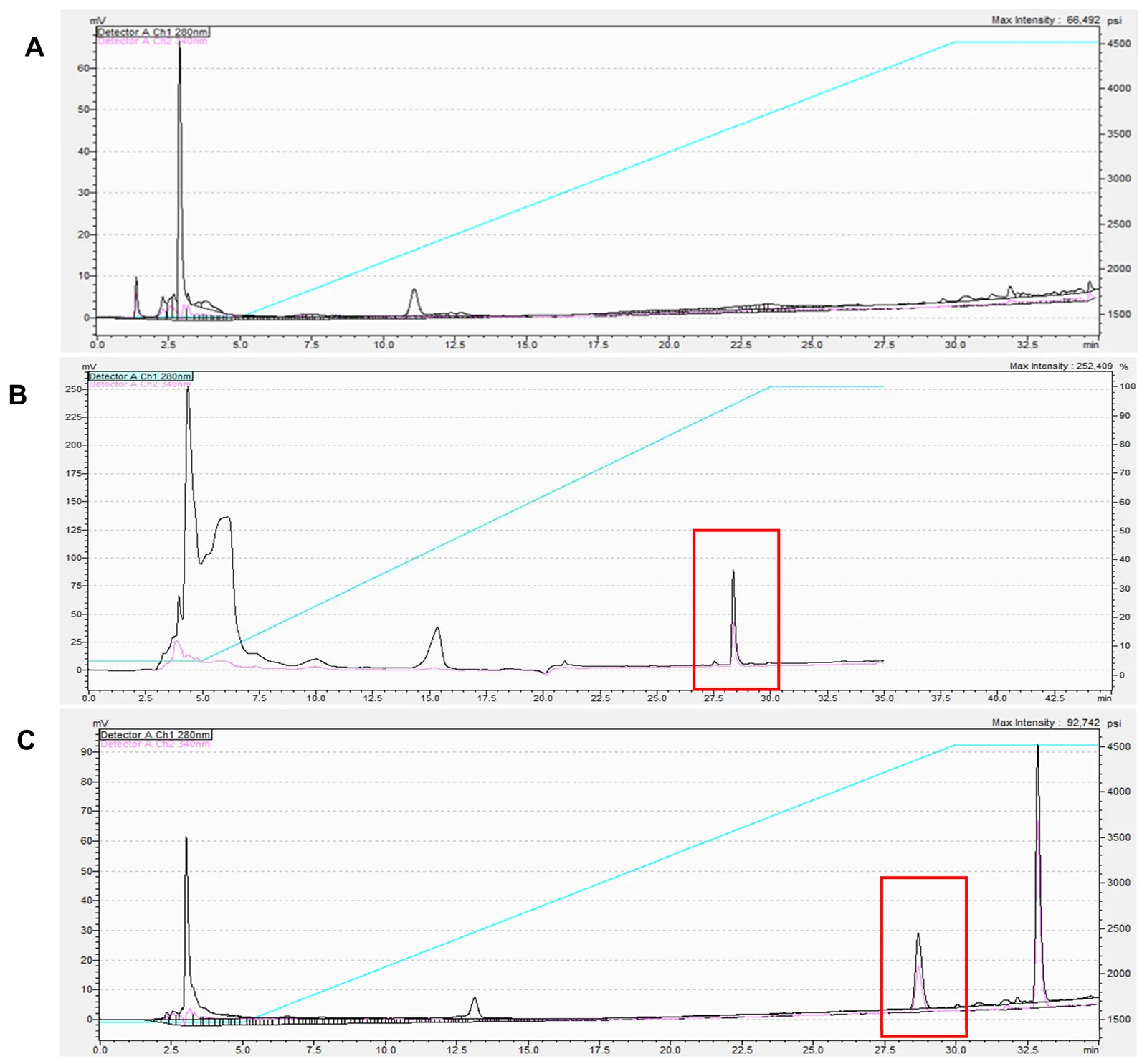
CXL remains detectable in serum following systemic administration. HPLC was performed on serum samples to evaluate the presence of CXL: (**A**) Chromatogram of untreated serum sample, serving as the negative control, shows no peak at the expected CXL retention time. (**B**) Chromatogram of serum sample spiked ex vivo with CXL displays a distinct peak (highlighted), confirming the identity and retention time of CXL. (**C**) Chromatogram of serum sample collected 6 h after in vivo administration of 10 mg/kg CXL shows a prominent peak (highlighted) at the same retention time, indicating systemic circulation of CXL at the time of collection. The black trace represents detector channel 1 (280 nm) and the pink trace represents detector channel 2 (340 nm), both plotted against the left axis (mV). The cyan trace represents system backpressure, plotted against the right axis (psi).

To evaluate whether CXL remained in systemic circulation, serum samples collected 6 h after intraperitoneal administration of 10 mg/kg b.w. CXL were analyzed by HPLC (Figure 9). Untreated serum showed no peak at the established CXL retention time (Figure 9A). Ex vivo addition of CXL to control serum generated a distinct peak at 29 min, confirming the retention time and peak identity of CXL (Figure 9B). A corresponding peak was detected in serum from CXL-treated mice (Figure 9C), confirming that CXL was present in systemic circulation 6 h after its administration.

### 3.9. CXL Is Detectable in Hepatic Tissue Following Systemic Administration

**Figure 10:**
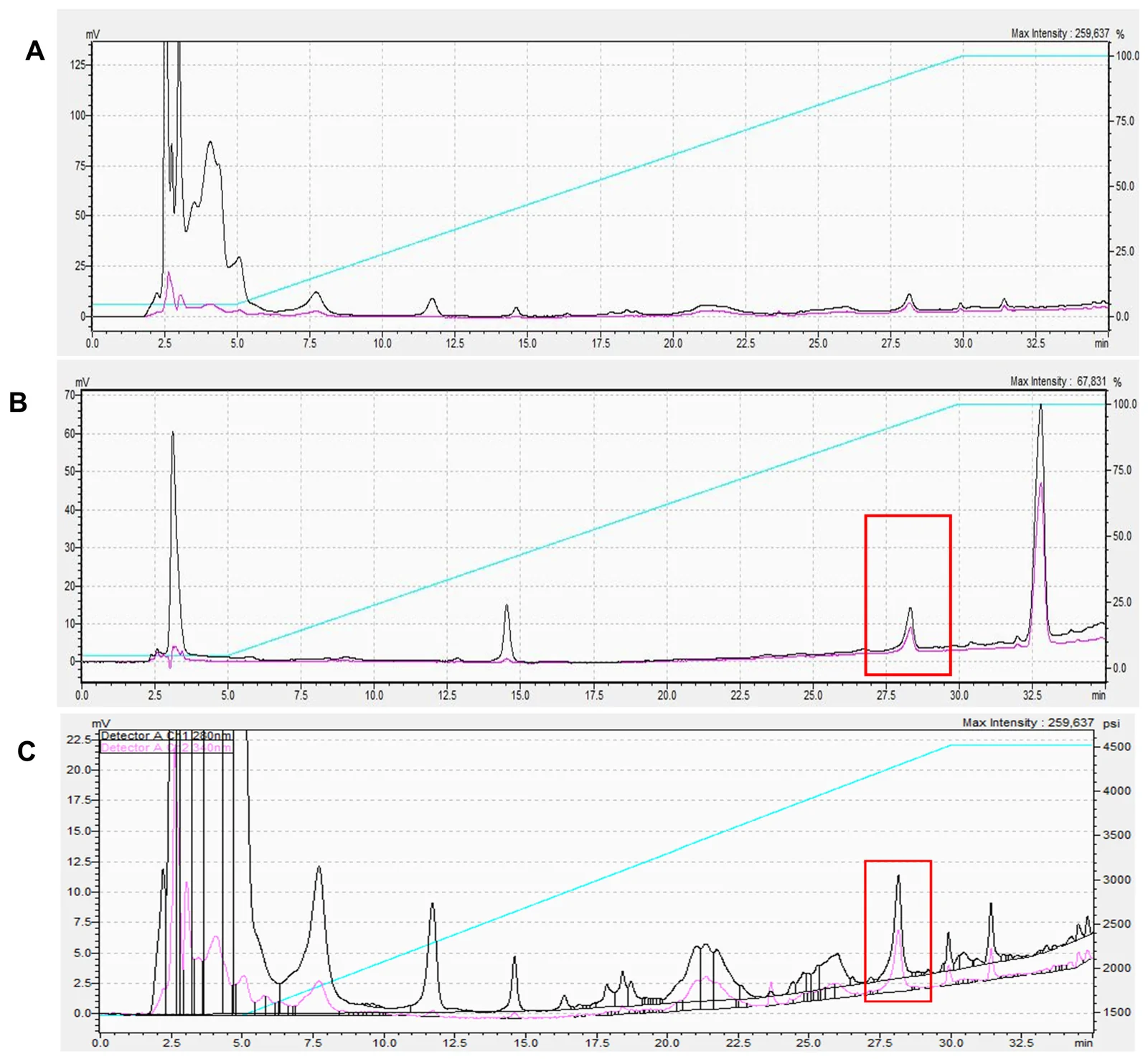
CXL is detectable in hepatic tissue following systemic administration. HPLC was performed on liver samples to evaluate the presence of CXL: (**A**) Chromatogram of untreated liver sample, serving as the negative control, shows no peak at the expected CXL retention time. (**B**) Chromatogram of liver sample spiked ex vivo with CXL displays a distinct peak (highlighted), confirming the identity and retention time of CXL. (**C**) Chromatogram of liver sample collected 6 h after in vivo administration of 10 mg/kg CXL shows a prominent peak (highlighted) at the same retention time, indicating the presence of CXL in hepatic tissue at the time of collection. The black trace represents detector channel 1 (280 nm) and the pink trace represents detector channel 2 (340 nm), both plotted against the left axis (mV). The cyan trace represents system backpressure, plotted against the right axis (psi).

To determine whether CXL was present in hepatic tissue, liver samples collected 6 h after intraperitoneal administration of 10 mg/kg b.w. CXL were analyzed by HPLC (Figure 10). Untreated liver homogenates showed no chromatographic peak at the established CXL retention time (Figure 10A). Ex vivo spike of control liver homogenates with CXL produced a distinct peak at the expected retention time of 29 min, confirming assay specificity and peak identity (Figure 10B). Liver tissue obtained from CXL-treated mice exhibited a peak at the corresponding retention time of 29 min (Figure 10C), demonstrating that CXL remained detectable in hepatic tissue under the experimental conditions.

## 4. Discussion

STING pathway plays a central role in innate immunity by mediating IFN-I production in response to cytosolic DNA. Consequently, pharmacological modulation of STING has become an area of considerable interest for the development of therapies targeting cancer, infectious diseases, and inflammatory disorders [3,4]. Despite a high degree of structural conservation between mSTING and hSTING, they exhibit substantial differences in ligand recognition, activation, and downstream signaling, leading to species-specific pharmacological responses. These species-dependent differences have important implications for preclinical drug development, as compounds that demonstrate promising activity in murine models may not exhibit comparable pharmacological behavior in humans [10].

In our previous work, we identified CXL as a non-nucleotide modulator of hSTING through MD-guided virtual screening. Subsequent computational, biophysical, and cellular studies demonstrated that CXL exhibits concentration-dependent effects on hSTING signaling and suggested that its activity is driven by modulation of receptor conformation and oligomerization [6]. The present study was undertaken to determine whether CXL exhibits comparable pharmacological behavior in the mSTING pathway.

Computational studies established a structural framework for evaluating CXL activity in mSTING. Site-restricted induced-fit docking predicted favorable binding of CXL within the cyclic dinucleotide binding pocket and yielded estimates consistent with experimentally reported binding affinities for DMXAA and 2′,3′-cyclic GMP–AMP (2,3-cGAMP) [14,15]. Although CXL and DMXAA occupied similar binding sites, subtle differences in their predicted interactions with the STING lid region suggested that CXL may induce distinct receptor conformations. These structural interpretations derive from molecular dynamics and induced-fit docking and are presented as proposed mechanisms pending experimental validation.

Our in vitro studies supported these computational predictions. Pre-treatment of 3T3-L1 cells with CXL enhanced DMXAA-induced STING, TBK1, and IRF3 phosphorylation and increased IFN-β mRNA expression compared with DMXAA alone. These findings indicate that CXL does not interfere with mSTING signaling but instead potentiates pathway activation. This contrasts with the inhibitory activity observed at low concentrations in hSTING and indicates that the functional outcomes differ from those previously reported for hSTING [6].

The in vivo findings closely paralleled the in vitro observations. Whereas serum IFN-β remained below the limit of quantification in untreated and vehicle-treated animals, it became quantifiable following DMXAA administration, consistent with DMXAA-induced activation of mSTING. CXL further enhanced DMXAA-induced IFN-β production in a non-monotonic manner, with maximal potentiation at 1 mg/kg b.w. and progressively smaller effects at 5 and 10 mg/kg b.w. This dose–response profile is consistent with the concentration-dependent behavior previously reported for CXL in hSTING [6]. Based on the computational modeling, we hypothesize that at lower concentrations CXL promotes initial receptor oligomerization and enhances DMXAA-induced signaling, whereas at higher concentrations, CXL may compete with DMXAA for the binding pocket, moderating the combined response. Together with the cellular data, these results indicate that CXL functions as a positive modulator of DMXAA-induced mSTING signaling in vivo, potentially by promoting a signaling-competent receptor state that augments the response to DMXAA.

The distinct functional behavior of CXL in mSTING and hSTING likely reflects intrinsic structural differences between the two receptors. Previous structural studies have demonstrated substantial differences in ligand recognition and conformational flexibility that account for the species-selective activity of compounds such as DMXAA. Despite a predicted interaction with mSTING and demonstrated binding to hSTING [6], CXL exhibits functional effects in the murine system that differ from those previously reported for hSTING. While CXL suppresses hSTING signaling, it appears to favor productive pathway activation in the mSTING model [6].

HPLC confirmed the presence of CXL in serum and liver six hours following its administration. In contrast, CXL was not detected in the kidney or spleen under the same analytical conditions. These experiments provide initial evidence that CXL reaches systemic circulation and remains available during the experimental period.

Finally, no treatment-related changes in body weight, food consumption, or general behavior were observed throughout the study. Histopathological examination of the liver, kidney, and spleen revealed preserved tissue architecture with no detectable treatment-associated abnormalities following combined CXL and DMXAA administration. These findings suggest that the treatment regimen employed in this study was well-tolerated.

Collectively, the agreement between computational modeling, cellular assays, and in vivo studies supports the conclusion that CXL acts as a positive modulator of DMXAA-induced mSTING signaling, in contrast to its previously reported inhibitory activity in hSTING [6]. These differences indicate that conventional mouse models may not accurately predict the effects of CXL on human STING signaling.

## 5. Limitations

These findings demonstrate acute in vivo activity of CXL following short-term treatment in a normal mouse model. STING dependency of the observed potentiation was not directly tested, and genetic approaches such as STING knockdown are planned in future studies. hSTING and mSTING were not compared under identical experimental conditions, as the human data derive from our previous work [6], and future studies will directly compare CXL activity in human and murine STING-expressing systems under matched conditions. Direct measurement of CXL binding to mSTING was not performed, and future studies will employ biophysical approaches such as surface plasmon resonance or microscale thermophoresis. CXL-alone conditions were not evaluated in the cellular or animal experiments, and future studies will characterize the activity of CXL in the absence of DMXAA. Tissue distribution was assessed qualitatively at a single time point, and future studies will apply quantitative methods across multiple time points to characterize the pharmacokinetics of CXL. IFN-β mRNA was assessed by end-point PCR, which provides a semi-quantitative measure of transcript abundance, and future studies will employ RT-qPCR for quantitative assessment. Only male mice were evaluated to determine the safety and efficacy of CXL, and future studies will evaluate the response to CXL in both male and female mice. Further studies using relevant disease models will be needed to evaluate its therapeutic efficacy under disease-specific conditions. Translation to humans in clinical trials may result in a different outcome. To evaluate this, in silico AI-driven measurements of efficacy, organ-on-chip, and patient-derived organoids may be performed.

## 6. Conclusions

This study demonstrates that CXL exhibits a distinct pharmacological profile in mSTING compared with hSTING. Computational modeling predicted favorable binding of CXL to mSTING, and in vitro and in vivo studies demonstrated enhanced STING pathway signaling following CXL treatment in combination with DMXAA. In addition, CXL was detectable in serum and liver after systemic administration, with no treatment-associated histological changes in liver, kidney, and spleen tissues. Collectively, these findings establish that CXL is well-tolerated under the conditions examined, remains systemically available, and acts as a positive modulator of DMXAA-induced murine STING signaling in vivo. The study underscores the importance of incorporating species-specific pharmacology and methodology when evaluating STING-targeted compounds for translation into human studies.

## Figures and Tables

**Table 1:** Experimental treatment groups and dosing schedule.

Treatment Group	Pre-Treatment	Treatment	*n*
1	none	none	6
2	CXL Vehicle Control	DMXAA Vehicle Control	6
3	CXL Vehicle Control	20 mg/kg DMXAA	6
4	1 mg/kg CXL	20 mg/kg DMXAA	6
5	5 mg/kg CXL	20 mg/kg DMXAA	6
6	10 mg/kg CXL	20 mg/kg DMXAA	6

## Data Availability

All data supporting the findings of this study are contained within the manuscript. Original images are provided in the Supplementary Materials.

## Supplementary Materials

The following supporting information can be downloaded at https://www.mdpi.com/article/10.3390/biology15211979/s1, Figure S1: Original images; Table S1: Individual animal serum IFN-β measurements and standard-curve data.

## Abbreviations

The following abbreviations are used in this manuscript:

STING	Stimulator of Interferon Genes
CXL	Clonixeril
hSTING	Human STING
mSTING	Murine STING
IFN-I	Type I interferons
cGAS	Cyclic GMP–AMP synthase
2,3-cGAMP	2′,3′-cyclic GMP–AMP
TBK1	TANK-binding kinase 1
IRF3	Interferon regulatory factor 3
MD	Molecular dynamics
RMSD	Root mean square deviation
RMSF	Root mean square fluctuation
CTD	Carboxy-terminus domain
