# Supplementary material for: Characterization of Clonixeril as a Positive Modulator of DMXAA-Induced Murine STING Signaling
Source: Biology (Basel). 2026 Nov 2;15(21):1979. doi: 10.3390/biology15211979 (PMC13761408; doi:10.3390/biology15211979)
Supplement: Supplementary file 1 [file biology-15-01979-s001.zip › Figure S1 (Original images).pdf]

Figure 3A

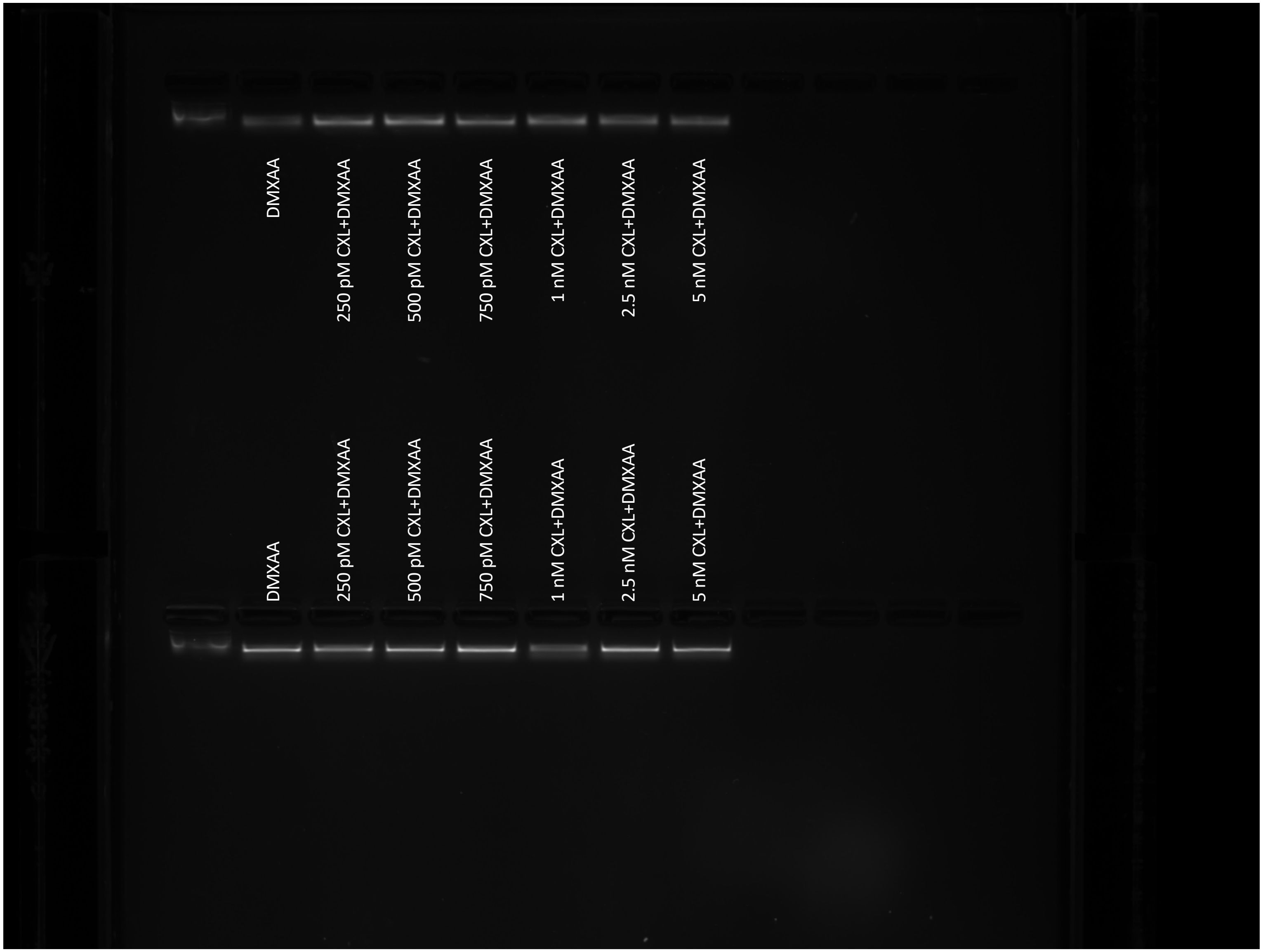
IFN- β
DMXAA
250 pM CXL+DMXAA
500 pM CXL+DMXAA
750 pM CXL+DMXAA
1 nM CXL+DMXAA
2.5 nM CXL+DMXAA
5 nM CXL+DMXAA
DMXAA
250 pM CXL+DMXAA
500 pM CXL+DMXAA
750 pM CXL+DMXAA
1 nM CXL+DMXAA
2.5 nM CXL+DMXAA
5 nM CXL+DMXAA
β – Actin

Figure 3B

IFN- β

β – Actin

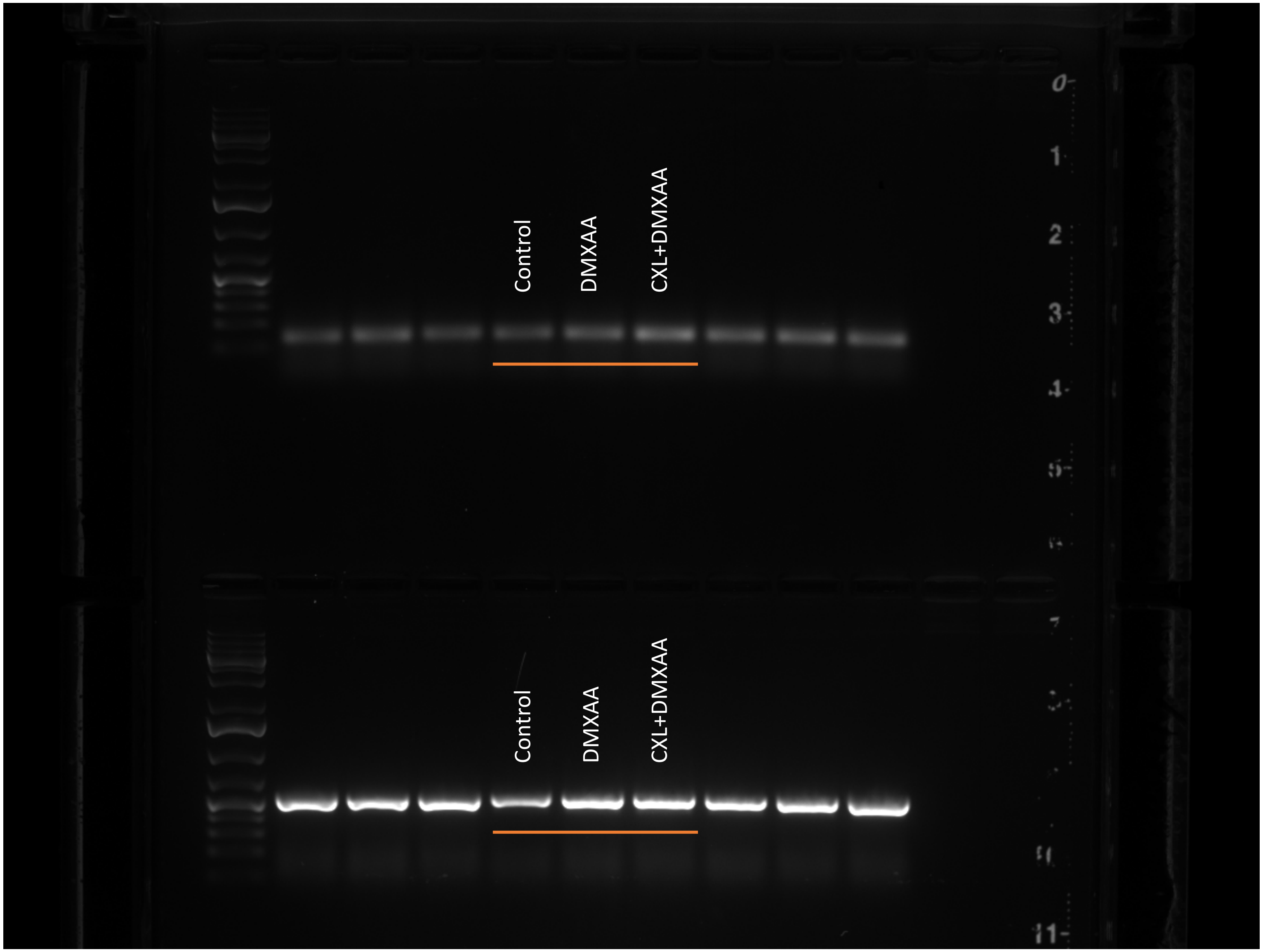

Figure 4A

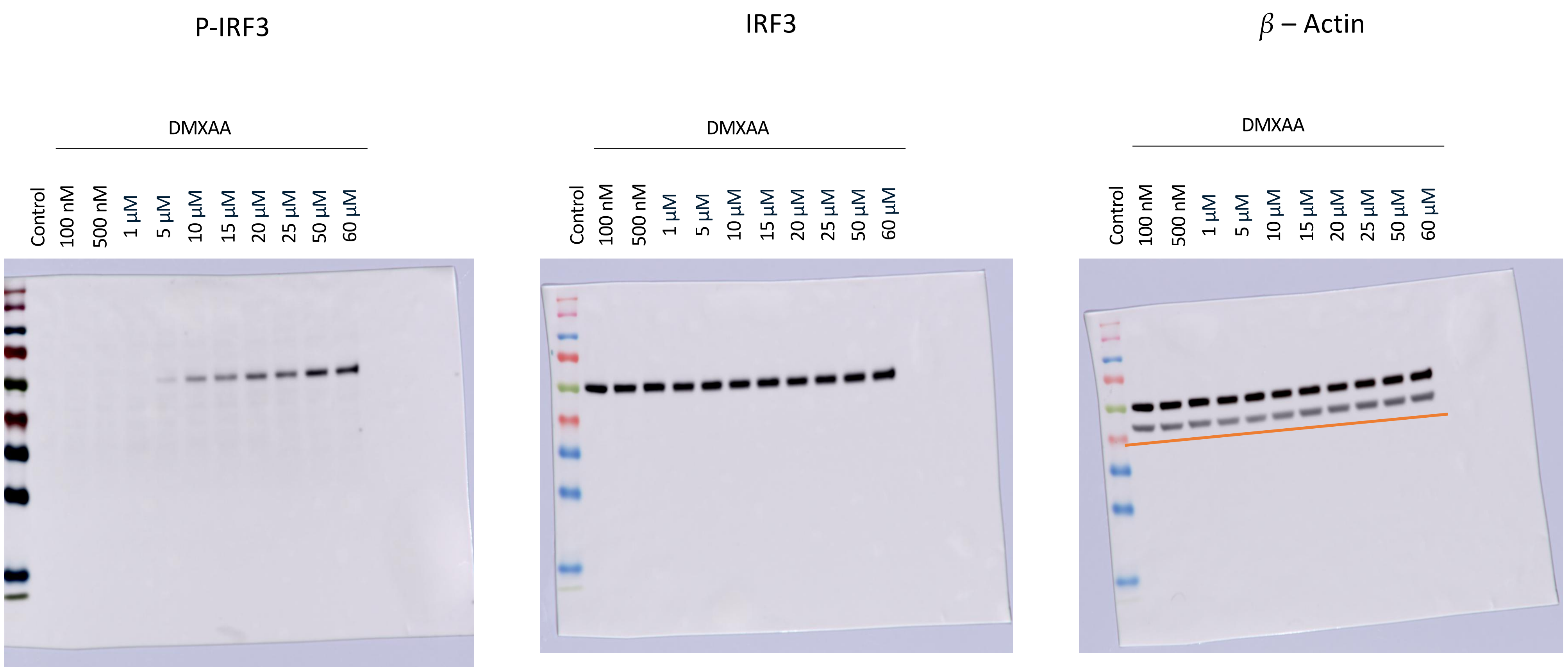

P-IRF3
DMXAA
Control
100 nM
500 nM
1 µM
5 µM
10 µM
15 µM
20 µM
25 µM
50 µM
60 µM
IRF3
DMXAA
Control
100 nM
500 nM
1 µM
5 µM
10 µM
15 µM
20 µM
25 µM
50 µM
60 µM
β – Actin
DMXAA
Control
100 nM
500 nM
1 µM
5 µM
10 µM
15 µM
20 µM
25 µM
50 µM
60 µM

Figure 4B

P-STING

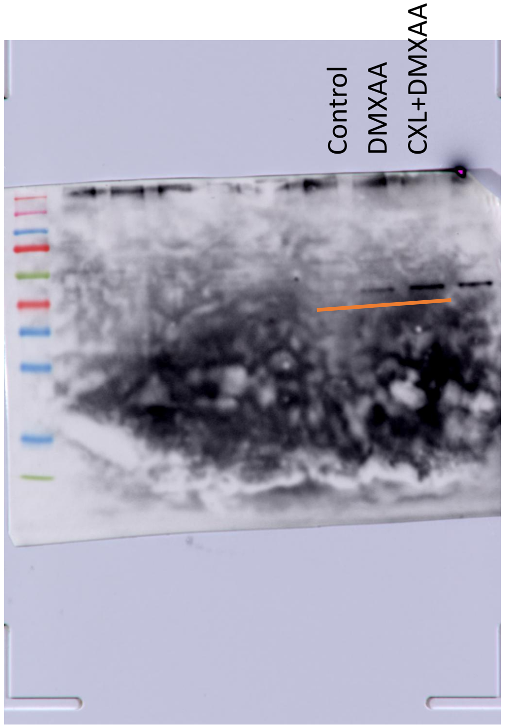

STING

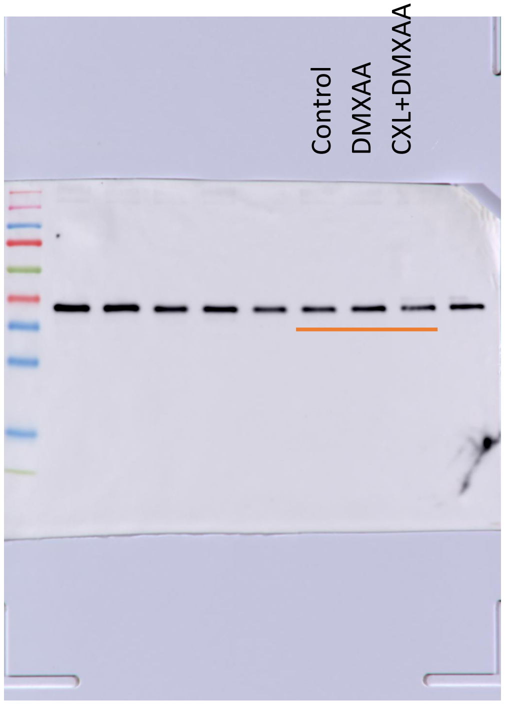

β – Actin

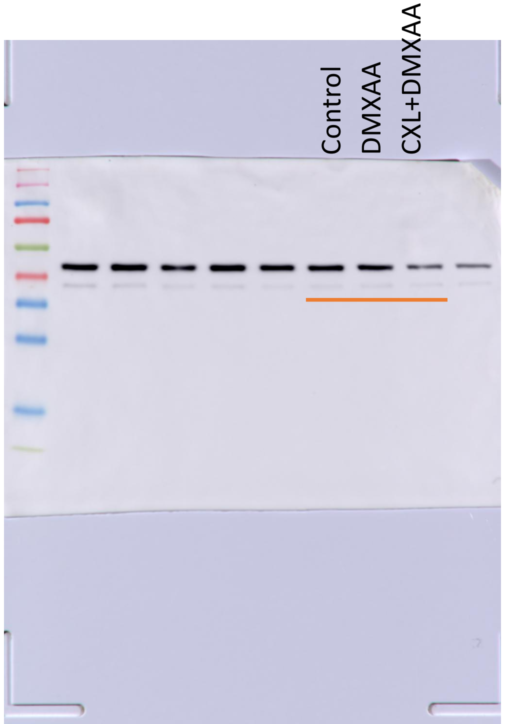

Figure 4C

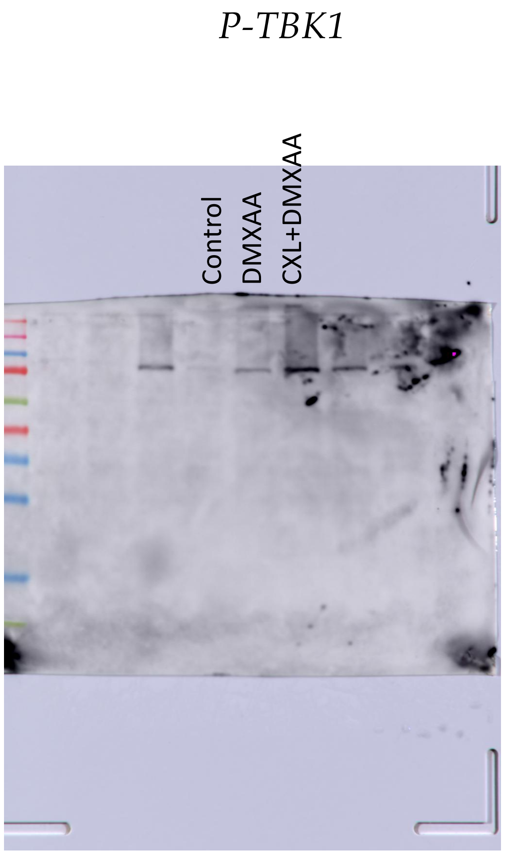
P-TBK1
Control
DMXAA
CXL+DMXAA

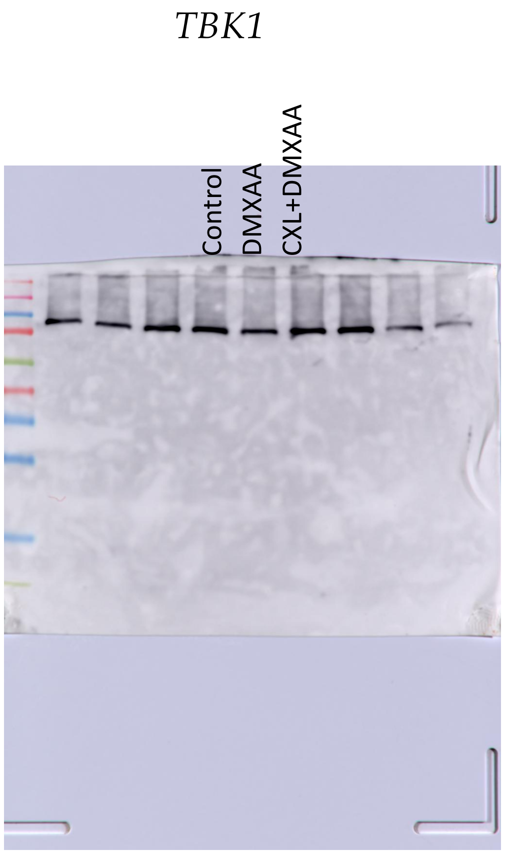
TBK1
Control
DMXAA
CXL+DMXAA

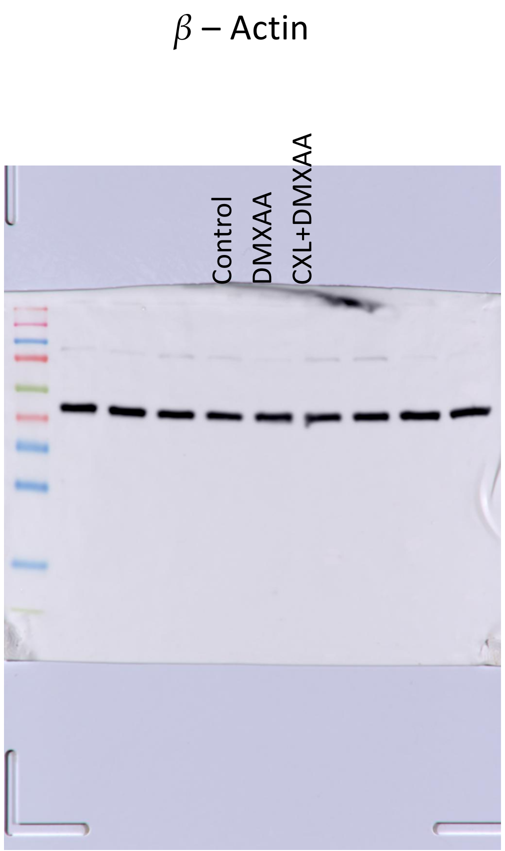
β – Actin
Control
DMXAA
CXL+DMXAA

Figure 4D

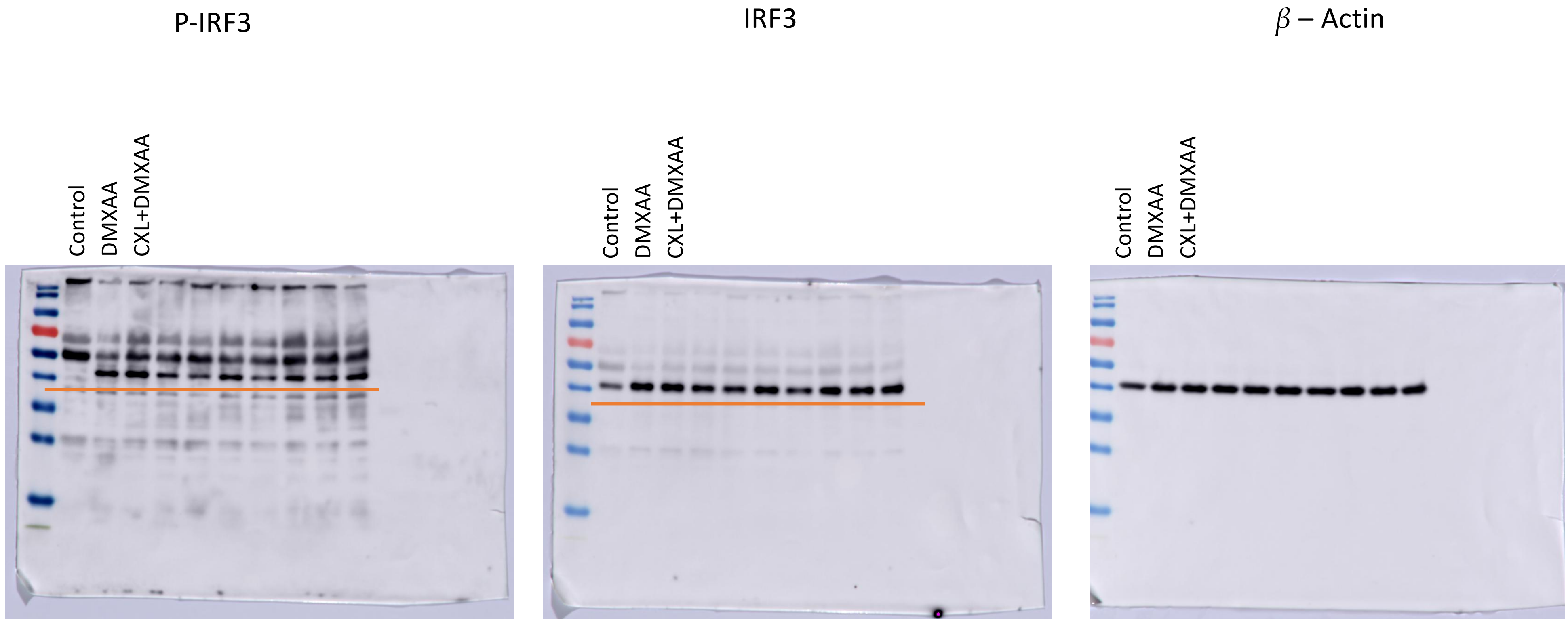
P-IRF3
Control
DMXAA
CXL+DMXAA
IRF3
Control
DMXAA
CXL+DMXAA
β – Actin
Control
DMXAA
CXL+DMXAA
